# Bacterial lysate add‐on therapy to reduce exacerbations in severe asthma: A double‐blind placebo‐controlled trial

**DOI:** 10.1111/cea.13990

**Published:** 2021-08-06

**Authors:** Geertje M. de Boer, Gert‐Jan Braunstahl, Esmee K. van der Ploeg, Cathelijne M. van Zelst, Alie van Bruggen, Guido Epping, Menno van Nimwegen, Gert Verhoeven, Erwin Birnie, Bianca M. Boxma‐de Klerk, Marjolein J. W. de Bruijn, Ralph Stadhouders, Rudi W. Hendriks, Gerdien A. Tramper‐Stranders

**Affiliations:** ^1^ Department of Pulmonary Medicine Franciscus Gasthuis & Vlietland Rotterdam The Netherlands; ^2^ Department of Pulmonary Medicine Erasmus University Medical Center Rotterdam The Netherlands; ^3^ Department of Cell Biology Erasmus University Medical Center Rotterdam The Netherlands; ^4^ Department of Pulmonary Medicine Maasstad hospital Rotterdam The Netherlands; ^5^ Department of Scientific Education Franciscus Gasthuis & Vlietland Rotterdam The Netherlands; ^6^ Department of Pediatrics Franciscus Gasthuis & Vlietland Rotterdam The Netherlands

**Keywords:** asthma, bacterial lysates, exacerbations, immune modulation, type 2 inflammation

## Abstract

**Background:**

Asthma exacerbations are frequently induced by respiratory tract infections (RTIs). Bacterial lysates have been described to possess immune‐modulatory effects and reduce RTIs as well as asthma symptoms in children. However, whether bacterial lysates have similar effects in adult asthma patients is unknown.

**Aims:**

To reduce asthma exacerbations by add‐on bacterial lysate therapy in adults with severe asthma and to characterize the clinical and immune‐modulatory effects of this treatment.

**Methods:**

Asthma patients (GINA 4) with ≥2 annual exacerbations in the previous year were included. The intervention regimen consisted of OM‐85/placebo for 10 consecutive days per month for 6 months during two winter seasons. Primary end‐point was the number of severe asthma exacerbations within 18 months. The study was approved by the national and local ethical review board and registered in the Dutch Trial Registry (NL5752). All participants provided written informed consent.

**Results:**

Seventy‐five participants were included (38 OM‐85; 37 placebo). Exacerbation frequencies were not different between the groups after 18 months (incidence rate ratio 1.07, 95%CI [0.68–1.69], *p* = 0.77). With the use of OM‐85, FEV1% increased by 3.81% (*p* = 0.04) compared with placebo. Nasopharyngeal swabs taken during RTIs detected a virus less frequently in patients using OM‐85 compared to placebo (30.5% vs. 48.0%, *p* = 0.02).

In subjects with type 2 inflammation adherent to the protocol (22 OM‐85; 20 placebo), a non‐statistically significant decrease in exacerbations in the OM‐85 group was observed (IRR = 0.71, 95%CI [0.39–1.26], *p* = 0.25). Immune‐modulatory effects included an increase in several plasma cytokines in the OM‐85 group, especially IL‐10 and interferons. Peripheral blood T‐ and B cell subtyping, including regulatory T cells, did not show differences between the groups.

**Conclusion:**

Although OM‐85 may have immune‐modulatory effects, it did not reduce asthma exacerbations in this heterogeneous severe adult asthma group. Post hoc analysis showed a potential clinical benefit in patients with type 2 inflammation.

## Graphical abstract

Seventy‐five severe asthma patients were included (38 OM‐85; 37 placebo). While intention to treat (ITT) analysis revealed no difference in exacerbation frequencies between the groups after 18 months, per protocol analysis in patients with type 2 inflammation (PPT2) showed a non‐significant decrease. Nasopharyngeal swabs detected a virus less frequently in the OM‐85 group during respiratory tract infections. Immune‐modulatory effects were seen in several plasma cytokines. To conclude, OM‐85 might be an effective add‐on therapy in severe asthma patients with type 2 inflammation.

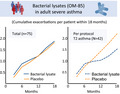

**Key Messages**

- OM‐85 did not reduce exacerbation frequency in the studied group of heterogeneous adult severe asthma patients;
- OM‐85 may be an effective add‐on therapy to reduce exacerbations in asthma patients with type 2 inflammation;
- Immune‐modulatory effects were observed with the use of OM‐85.

## 1 INTRODUCTION

Asthma patients with recurrent exacerbations suffer the highest disease burden and account for over half of the asthma‐associated healthcare expenditure, with 80% of the total direct costs of asthma attributed to the treatment of exacerbations.^1^ About 50% of exacerbations are elicited by viral respiratory tract infections (RTIs).^2^, ^3^ Therefore, the prevention of RTIs is critical for reducing exacerbation frequencies.^4^ In several European and Asian countries, bacterial lysates have been used for the prevention of recurrent RTIs since the early 1950s.^3^, ^5^, ^6^, ^7^ Bacterial lysates are non‐viable bacterial extracts obtained by either chemical or mechanical cellular lysis of bacterial cultures and lyophilization.^5^, ^8^, ^9^, ^10^ Although the most well‐known bacterial lysate OM‐85 has shown clinical efficacy in preventing RTIs, the immune‐modulatory effects of OM‐85 in humans are still not fully elucidated.^5^, ^7^, ^11^, ^12^, ^13^ Murine studies suggest that dendritic cell (DC) activation in gut‐associated lymphoid tissue results in local and systemic stimulation of antiviral cytokine production, including Th1 cytokines such as interferons, as well as local immunoglobulin secretion.^14^, ^15^ Asthma is a phenotypically heterogeneous disease, mainly driven by type 2 inflammation. In murine asthma studies, a decrease in pulmonary Th2 cytokines was observed after OM‐85 treatment.^10^, ^14^, ^15^, ^16^ Additionally, OM‐85 induced pulmonary recruitment of regulatory T cells along with a decrease in bronchial hyper‐reactivity in animal models.^15^ Some studies in children suggest shifts in the Th1/Th2 cytokine balance.^17^, ^18^

Several recent reports and meta‐analyses concluded bacterial lysate therapy has a favourable safety profile and reduced RTIs, COPD exacerbations and preschool wheezing episodes.^5^, ^7^, ^11^, ^12^, ^13^, ^18^ Also, a recent study showed bacterial lysates to be effective in the improvement of the clinical course of allergic rhinitis.^19^ There is some evidence for a decrease in asthma symptoms in school children.^17^, ^18^, ^20^ In contrast, studies of adult patients with asthma are scarce. Therefore, our aim was to investigate the potential of add‐on bacterial lysate therapy to reduce asthma exacerbations in adults with severe asthma and to characterize the clinical, microbiological and immune‐modulatory effects of this treatment.

## 2 PATIENTS AND METHODS

### 2.1 Study design and setting

We conducted a 1:1 double‐blind, randomized, placebo‐controlled study in two large teaching hospitals in Rotterdam, the Netherlands, between September 2016 and April 2019. The study was approved by the national and local ethical review board and registered in the Dutch Trial Registry (NL5752). All participants provided written informed consent.

### 2.2 Participants

Asthma patients between 16 and 60 years of age with ≥2 physician‐diagnosed asthma exacerbations (treated with oral corticosteroids and/or antibiotics) in the previous year were invited to participate in this study. Asthma diagnosis was based on the GINA 2016 criteria.^21^ Other inclusion criteria were as follows: asthma control questionnaire (ACQ) score >0.75 and optimal maintenance medication according to GINA 4 (medium/high‐dose inhaled corticosteroid and long‐acting β_2_‐agonists). Exclusion criteria were as follows: other relevant respiratory conditions; systemic immunological diseases and/or immunosuppressive medication (including all biologicals); untreated comorbidities; known non‐compliance; current or planned pregnancy; non‐comprehension of Dutch language; and current or past smoking with >10 pack years. Type 2 inflammation (T2^+^) was defined according to the GINA 2020 guidelines (blood eosinophils ≥0.15*10^9^/L and/or fractional exhaled nitrogen oxide (FeNO) ≥20 ppb and/or presence of a clinical relevant allergy).^22^ During the study, patients were treated by their own pulmonary physician for their asthma, according to local protocols.

### 2.3 Intervention

Patients received 7 mg capsules of either OM‐85 or matching placebo (Broncho‐Vaxom, OM Pharma) in addition to their asthma treatment regimen. OM‐85 consisted of an extract of lyophilized lysates from 21 strains from the following pathogenic bacteria: (1) *Haemophilus influenzae*; (2) *Streptococcus pneumoniae*, *sanguinis and pyogenes*; (3) *Klebsiella pneumoniae* and ‐*ozaenae*; (4) *Staphylococcus aureus* and (5) *Moraxella catarrhalis*.^23^ Capsules were to be taken on 10 consecutive days monthly, starting either the 1st or the 15th of each month, during 6 months for 2 winter seasons starting in 2016 or 2017 (October until March; 2016/17 and 2018/2019). To monitor medication adherence, patients returned strips and packaging. Remaining drugs were destroyed.

### 2.4 Study protocol

**FIGURE 1:**
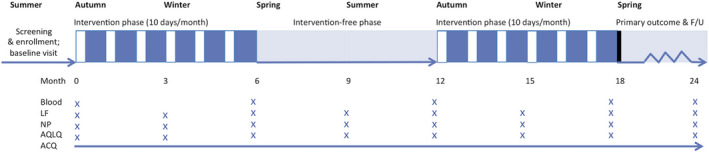
Study outline: Visits were scheduled every 3 months and at the time of an exacerbation. ACQ, asthma control questionnaire; AQLQ, asthma quality of life questionnaire; LF, lung function; NP, nasopharyngeal swab

The protocol included 3 monthly visits at baseline (*T* = 0) and thereafter at 3, 6, 9, 12, 15 and 18 months (study end) and exacerbation visits (Figure 1). During these visits, we assessed clinical condition and asthma control and took a nasopharyngeal swab. Every 6 months, blood was drawn. During asthma exacerbations, additional nasopharyngeal swabs and blood were collected when the patient presented at the hospital. During common colds, patients were asked to collect a nasopharyngeal swab at home. Participants received a weekly digital questionnaire addressing ACQ, number of RTIs and exacerbations. Participants that stopped prematurely with the study, were included in the analysis of the primary end‐point after consent.

### 2.5 Study outcomes

The primary study outcome was the number of asthma exacerbations within the first 18 months after start of intervention. An asthma exacerbation was defined as physician‐diagnosed worsening of asthma symptoms with the need for emergency treatment with oral corticosteroids. Asthma exacerbations were treated with oral corticosteroids (40 mg once daily for 5 days) and antibiotics if necessary, according to the treating physician. Secondary study outcomes were as follows: time to first and second asthma exacerbation, number of viral RTIs, prescriptions of oral corticosteroids and/or antibiotics, ACQ and asthma‐related quality of life (AQLQ), pulmonary function, FeNO and immunological measurements. Also, adverse events were registered.

### 2.6 Sample size calculation

Literature on the use of OM‐85 for exacerbation prevention in adults with asthma was unavailable. Power analysis was targeted at a 20% reduction in asthma exacerbations, based on previous literature describing a 20%–40% reduction of RTIs in children and adults.^24^, ^25^ Two x 36 patients were required to have 80% chance of detecting the 20% decrease in asthma exacerbations measured at a 5% significance level (two‐sided, assumed participants having on average 2 ± 0.6 exacerbations per year based on pilot data).

### 2.7 Randomization and treatment allocation

Block randomization of all patients was stratified for atopy status (defined as history of allergic symptoms and serum levels of specific IgE for respiratory allergens >0.7 KU/L). Randomization was performed by allocating the first consecutive number in the strata to the participant. Patients, investigators, treating physicians and the hospital pharmacists were blinded to the allocation during the study. The unblinding key was released after all active participants finished their 18‐month visit and after the exacerbations of every participant had been filed in the study database were verified by a second investigator, and the database was locked. The investigational active and matching placebo medicinal products were packed‐ and labelled by OM Pharma and shipped directly to the hospital trial pharmacy. Medication was handed over to patients in packages with 3 strips of 10 capsules.

### 2.8 Lung function, microbial and immunological analyses

Forced expiratory volume in 1 s as percentage of predicted (FEV1%) was performed with the Vmax Sensor Medics Viasys, type 6200 Encore^26^ and FeNO was measured with the Niox‐Flex (Aerocrine AB).^27^

Nasopharyngeal swab liquid was stored at −80°C and analysed batch‐wise for the presence of respiratory viruses and *Mycoplasma pneumoniae* by multiplex PCR^28^ for baseline, 3‐, 6‐month and exacerbation samples.

Blood leukocyte differentiation, serum IgE, IgA, IgM and IgG were measured with Beckman Coulter equipment (DxH 800, Fabia and Immage 800) for baseline, 6‐, 12‐ and 18‐month samples.

Plasma cytokines IL‐6, IL‐8, IL‐9, IL‐10, IL‐13, IL‐17E, IL‐17F, TNFα, IFNγ and IFNλ were quantified with ELISA (Duosets, R&D Systems) for baseline, 6 and 12‐month samples. Plasma cytokines measured below the lower detection limit (D) were set to √D.

Peripheral blood mononuclear cells (PBMC) phenotyping by flow cytometry was performed for baseline, 6‐, 12‐ and 18‐month samples of T2^+^ participants being adherent to the study protocol (PPT2). Supplementary Methods show the flow cytometric protocol; Table S1 lists antibodies used for PBMC phenotyping; Figure S3 shows the gating strategies for CD4^+^ and CD8^+^T cells and intracellular cytokines, regulatory T cells and B cells.^29^

### 2.9 Pre‐specified statistical analysis

Pre‐specified statistical analysis was performed for all included patients. Study data were analysed according to the intention to treat (ITT) principle. A negative binomial regression model with number of exacerbations as dependent variable and allocated intervention (OM‐85/placebo) as independent variable, adjusted for age and atopy was used to estimate the primary outcome, number of exacerbations within 18 months; missing data were excluded from analysis. Adjusted outcomes are presented as incidence rate ratio (IRR) with 95% confidence intervals (95%CI) of the binomial regression.

Analysis of secondary end‐points was only adjusted for atopy (stratification variable), as age in both groups was comparable. Time to first and second exacerbation was evaluated using Kaplan‐Meier curves with logrank test and Cox regression. Repeated measurements of continuous variables were used to compare differences within patients over time (trends over time) and differences between patients over time (treatment effect) by linear mixed modelling (covariance: unstructured; fixed effects: time, intervention (placebo/OM‐85) and the covariate atopy status), resulting in estimated marginal differences of continuous secondary end‐points. The estimated difference (ED) between both groups is reported with the corresponding 95%CI and *p*‐value. Also, median values and interquartile ranges of the data are shown. For cytokine data, to prevent under‐ or overestimation, outlier analysis was performed by clipping the upper and lower 10% of data points within groups. Skewed variables were log‐transformed before repeated measurements were performed. Fold‐changes were used to compare differences in time between groups for plasma cytokines (baseline vs. 12 months), with the Mann‐Whitney *U* test, as data were non‐parametric. A *p* < 0.05 (two‐sided) was regarded statistically significant. To correct for multiple‐testing Bonferroni correction was applied for fold‐change data resulting in *p* < 0.005 for statistical significance. All analyses were performed using SPSS 26.0 (IBM) and GraphPad 8.4.3. Interim analysis was not performed.

### 2.10 Post hoc analysis

**FIGURE 2:**
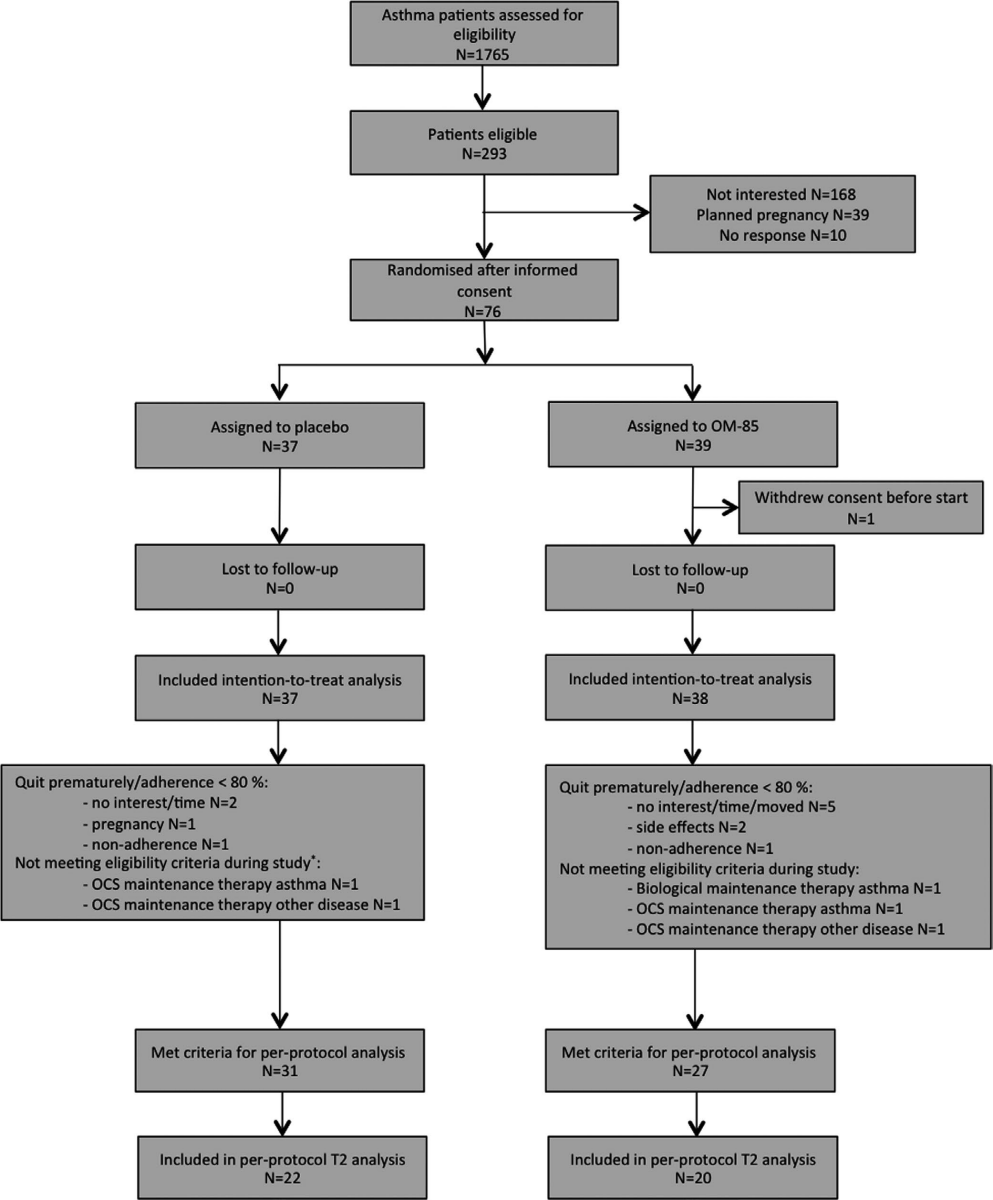
Flow diagram of inclusion. N, number; OCS, oral corticosteroids; T2, type 2 inflammation

Post hoc subgroup analyses, with identical outcomes and statistical plan as described above, were performed for patients being adherent to the study protocol (PP) and PPT2 (*N* = 42). Rationale of this PPT2 analysis is that bacterial lysate therapy decreases childhood asthma exacerbations, in asthma that often is allergic in origin.^17^, ^18^, ^20^ Patients in the PP analysis were either found to be non‐adherent or expressed no interest/time anymore during the study. Also, patients that started a biological or systemic corticosteroids during the first 18 months of this study were excluded from the PP analysis based on protocol violation (Figure 2).

## 3 RESULTS

Of 293 eligible patients, 75 patients provided informed consent and participated in this study (*n* = 38 OM‐85 vs. *n* = 37 placebo) (Figure 2).

Placebo and OM‐85 groups showed comparable age, gender, ACQ, FEV1% and asthma exacerbations (Table 1). Adherence, as monitored by questionnaires and returned study medication packages, was not different between the two groups (Figure 2).

**TABLE 1:** Participant demographics

	OM−85 (*N* = 38)	Placebo (*N* = 37)
Clinical variables		
Age	40.00 [28.0–51.3]	41.0 [31.5–54.5]
Body Mass Index	28.0 ± 5.5(18.5–42.3)	28.7 ± 6.4 (20.0–49.0)
Sex (female)	31 (81.6)	30 (81.1)
Pack years	0.00 [0.0–4.0]	0.00 [0.00–4.5]
ACQ	2.09 [1.3–2.7]	2.00 [1.0;2.8]
AQLQ	5.1 ± 1.0 (2.7–6.9)	5.0 ± 1.0 (3.2–6.9)
Household with children	19 (50.0)	21 (56.8)
Exacerbations/yr before study	2.5 ± 0.9 (2–6)	2.7 ± 1.2 (2–8)
FEV1%pred	90.39 ± 15.48 (55.8–118.9)	86.81 ± 13.55 (53.10–108.90)
FeNO (ppb)	14.5 [11.0–22.0]	16.0 [11.0–32.5]
Beclometason dipropionate equivalent ICS (µg/day)	1000.0 [1000.0;2000.0]	1000.0 [950.0;2000]
Asthma phenotype		
Type 2 inflammation^a^	28 (73.7)	27 (73.0)
Atopy^b^	25 (65.8)	22 (59.5)
Childhood onset asthma	20 (52.6)	24 (64.9)

Note

Data shown in mean ± SD (min‐max). Median [25th‐75th] or absolute *N* (%) counts.

Abbreviations: ACQ, asthma control questionnaire; AQLQ, asthma quality of life questionnaire; yr, year; FEV1%pred, forced expiratory volume in 1 second percentage of predicted; FeNO, forced expiratory nitrogen oxide; ICS, inhaled corticosteroids.

^a^ Type 2 inflammation defined as blood eosinophils ≥0.15*10^9^/L and/or forced expiratory nitrogen oxide (FeNO) ≥20 pp;

^b^ Atopy defined as history of allergic symptoms and serum levels of specific IgE for respiratory allergens >0.7 KU/L.

### 3.1 Primary outcome; intention to treat

**FIGURE 3:**
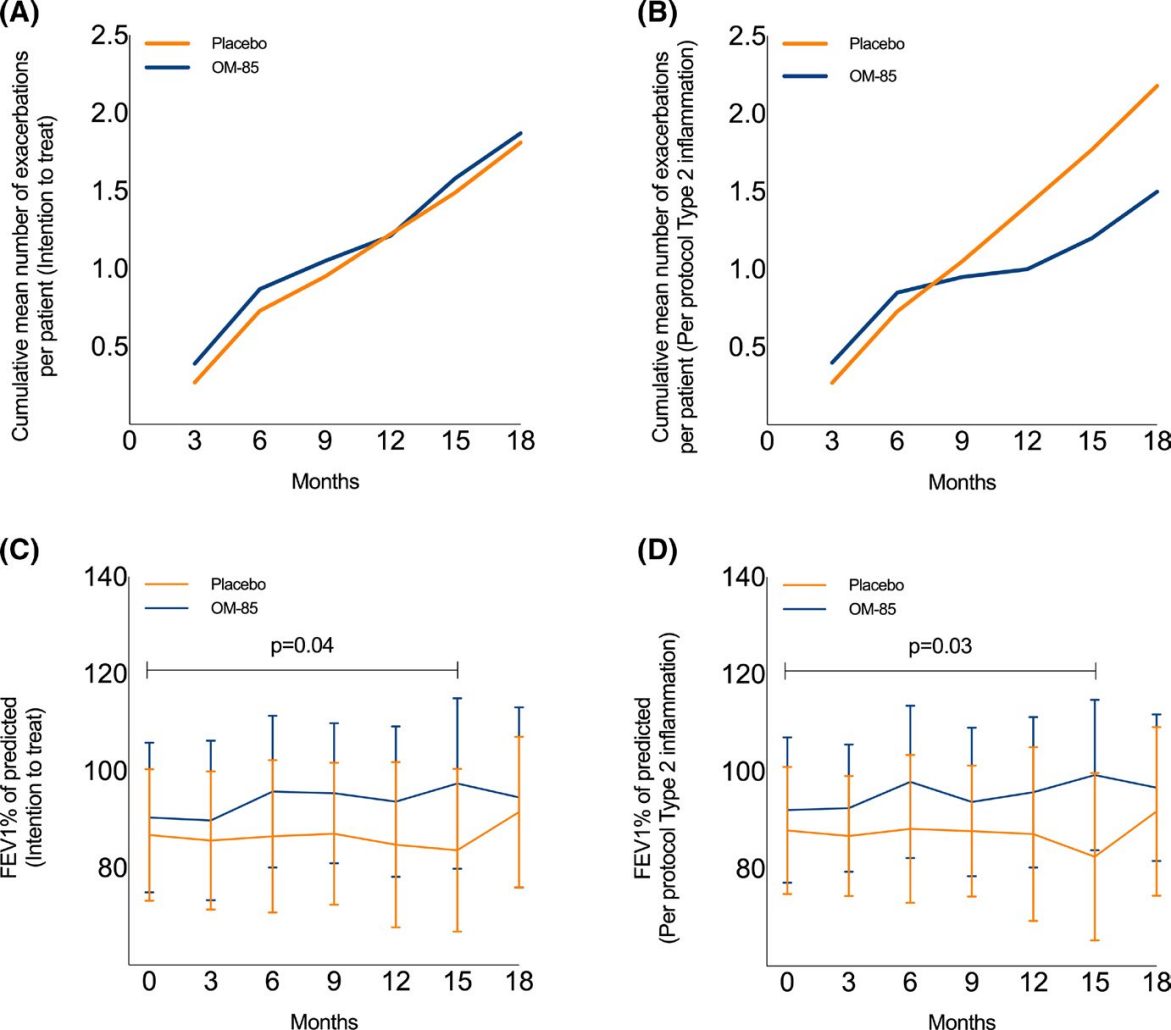
Clinical outcomes after OM‐85 and placebo treatment: (1) Cumulative mean number of exacerbations per patients during the study: (A) patients included in the intention to treat analysis (mean exacerbations at 18 months: 1.87 ± 1.71 vs. 1.81 ± 1.91, IRR 1.07. 95% CI [0.68–1.69]. *p* = 0.77); (B) patients with type 2 inflammation included in the per protocol analysis (mean exacerbations at 18 months: 1.50 ± 1.50 vs. 2.18 ± 1.94 IRR 0.71CI [0.39–1.26]. *p* = 0.25). 2) Percentage of forced expiratory volume in 1 second trend in time: (C) patients included in intention to treat analysis (ED between groups in time 3.81%, 95%CI [0.17;7.46], *p* = 0.04) and (D) patients with type 2 inflammation included in the per protocol (ED between groups in time 5.38%, 95%CI [0.67;10.09], *p* = 0.03

The cumulative number of asthma exacerbations over the 18 months period was 71 (mean number per patient: 1.87 ± 1.71) in the OM‐85 group and 67 (mean number per patient: 1.81 ± 1.91) in the placebo group (IRR) 1.07, 95%CI [0.68–1.69], *p* = 0.77) (Figure 3a). Mean number of asthma exacerbations within the first 6 months, months 6–12 and months 12–18 were also not different between the two groups (Table S2). A separate analysis of patients <40 years of age and >40 years of age did not show differences between OM‐85 and placebo groups.

### 3.2 Secondary outcomes; intention to treat

Time to first exacerbation or between first and second exacerbation was comparable for both groups (logrank *p* = 0.610 and *p* = 0.560 respectively). Cox‐regression analysis showed a hazard ratio of 1.09 for OM‐85 (95%CI 0.63–1.91, *p* = 0.75) (Figure S1a, b). Medication use for severe exacerbations was identical between OM‐85 and placebo (data not shown). Total number of adverse events did not differ between OM‐85 and placebo (11 vs. 6, *p* = 0.29) (Table 2).

**TABLE 2:** (Severe) adverse events during the complete study period

	OM‐85 (*N* = 38)	Placebo (*N* = 37)
Total number of patients with adverse events (number of serious adverse events)	11 [2]	6 [2]
Respiratory (dyspnoea/RTI)	3 [1]	1 [1]
Gastro‐intestinal (abdominal pain, diarrhoea, nausea)	4	1
Neurological (headache, dizziness, fatigue)	1	0
Skin (urticaria, rash)	0	2
Other	0	1
Multiple	3 [1]	1 [1]

Note

Total number of serious adverse events between brackets.

ACQ and AQLQ improved for all participants during the study, with no additional effect of OM‐85 (Table 3). FEV1% increased over time in patients using OM‐85 compared with placebo (ED 3.81%, 95%CI [0.17;7.46], *p* = 0.04) (Figure 3c; Table 3).

**TABLE 3:** Secondary end‐points for patients included in the intention to treat analysis (*N* = 75)

	Linear Mixed Model			OM‐85			Placebo		
	ED^a^	95% CI	*p*	T0	T6	T12	T0	T6	T12
Clinical parameters									
ACQ	0.19	0.23–0.62	0.36	2.06 ± 0.84	1.75 ± 1.09	1.70 ± 0.98	1.98 ± 1.09	1.18 ± 0.76	1.33 ± 0.83
AQLQ	0.05	−0.25;0.36	0.79	5.12 ± 1.05	5.51 ± 0.73	5.51 ± 0.83	5.03 ± 1.03	5.41 ± 0.86	5.38 ± 0.86
FEV1*	3.81	0.17;7.46	0.04	86.81 ± 13.55	103.84 ± 81.75	84.79 ± 17.03	90.39 ± 15.48	98.36 ± 15.54	93.72 ± 15.5
FeNO	2.81	−11.88;17.51	0.70	31.51 ± 43.91	30.03 ± 36.43	31.71 ± 39.79	27.72 ± 31.17	28.64 ± 28.21	26.35 ± 25.88
Blood and serum routine immunological measurements									
Eo	−0.07	−42;0.28	0.69	0.26 ± 0.28	0.29 ± 0.26	0.26 ± 0.28	0.25 ± 0.23	0.24 ± 0.19	0.27 ± 0.28
Neutro	−0.04	−0.19;0.11	0.57	4.33 ± 1.83	4.17 ± 1.78	3.88 ± 1.29	4.39 ± 1.94	4.03 ± 1.17	4.46 ± 1.86
IgG	0.01	−0.11;0.12	0.94	9.44 ± 1.85	9.69 ± 2.15	10.05 ± 2.40	9.78 ± 2.59	9.83 ± 2.71	9.73 ± 2.64
IgA	0.12	−0.11;0.36	0.30	2.05 ± 1.07	2.18 ± 1.19	2.20 ± 1.25	2.07 ± 1.26	1.91 ± 2.26	1.92 ± 1.02
IgM	0.15	−0.09;0.39	0.22	1.23 ± 0.59	1.18 ± 0.52	1.18 ± 0.52	1.30 ± 1.51	1.03 ± 0.66	1.07 ± 0.60
IgE	0.26	−0.51;1.04	0.50	300.35 ± 449.89	300.49 ± 436.23	365.97 ± 529.82	387.24 ± 792.09	380.29 ± 777.45	412.91 ± 850.94
Plasma cytokine measurements by ELISA									
IL‐6*	0.67	0.07;1.28	0.03	1.94 [0.77–14,99]	2.24 [0.77–9.52]	3.06 [0.77–9.44]	0.77 [0.77–3.64]	0.77 [0.77–3.23]	0.77 [0.77–2.09]
IL‐8	−0.02	−0.30;0.26	0.88	1.40 [1.40–1.40]	1.40 [1.40–1.40]	1.40 [1.40–1.40]	1.40 [1.40–1.40]	1.40 [1.40–1.40]	1.40 [1.40–1.40]
IL‐9	0.22	−0.11;0.56	0.19	2.21 [2.21–2.21]	2.21 [2.21–2.21]	2.21 [2.21–6.75]	2.21 [2.21–2.21]	2.21 [2.21–2.21]	2.21 [2.21–2.21]
IL‐10*	0.38	0.09;0.66	0.01	2.71 [1.40–28.31]	3.53 [1.40–34.15]	4.11 [1.40–30.28]	1.40 [1.40–6.62]	1.40 [1.40–3.45]	1.40 [1.40–2.70]
IL‐13*	0.59	0.09;1.09	0.02	151.82 [5.85–138.80]	265.24 [74.97–828.29]	335.38 [89.08–1036.40]	6.85 [6.85–393.93]	6.85 [6.85–370.94]	6.85 [6.85–152.81]
IL‐17E*	0.40	0.06;0.75	0.02	17.12 [7.83–35.88]	15.60 [7.67–27.92]	22.65 [9.19–35.78]	8.32 [1.91–18.28]	4.81 [1.91–20.10]	1.91 [1.91–15.99]
IL‐17F	0.32	−0.05;0.69	0.09	16.97 [3.82–43.78]	18.83 [11.33–33.87]	19.56 [8.77–30.27]	10.45 [2.50–32.47]	7.59 [2.50–28.44]	8.69 [2.50–23.03]
IFNγ	0.43	−0.24;1.11	0.20	4.48 [1.53–9.81]	5.86 [2.12–10.22]	5.86 [2.15–11.07]	5.39 [1.53–9.57]	4.99 [1.53–10.33]	5.09 [1.53–6.90]
IFNλ	0.18	−0.11;0.46	0.22	20.78 [5.59–440.08]	5.59 [5.59–512.06]	118.58 [5.59–618.19]	5.59 [5.59–5.59]	5.59 [5.59–117.21]	5.59 [5.59–5.59]
TNFα	0.07	−0.56;0.71	0.82	2.79 [2.79–14.64]	2.79 [2.79–16.56]	2.79 [2.79–33.17]	2.79 [2.79–2.79]	2.79 [2.79–10.61]	2.79 [2.79–2.79]

Abbreviations: ACQ, asthma control questionnaire; AQLQ, asthma quality of life questionnaire; Eo, eosinophils (expressed as ×10^9^/L); FEV1, forced expiratory volume in 1 second, expressed as %; IFN, interferon (expressed as pg/ml); Ig, immunoglobulin (IgG, IgA and IgM expressed as g/L, IgE expressed as IU/ml; IL, interleukin (expressed as pg/ml); Neutro, neutrophils (expressed as ×10^9^/L); p, p‐value; TNF, tumour necrosis factor (expressed as pg/ml).

* Significant difference.

### 3.3 Microbiological and immunological analysis; intention to treat

We next compared relevant microbiological and immunological parameters between the two treatment groups. At baseline, 3 and 6 months, the proportion of samples in which viruses could be detected did not differ (Figure S2). In total, 329 unique RTI’s were reported within the weekly questionnaires by 66 participants during the complete study period (165 OM‐85 vs. 164 placebo). Home‐collected nasopharyngeal swabs were handed in from 61 RTIs, 23 (38%) contained a detectable virus by PCR (OM‐85 30.5% vs. placebo 48.0%, *p* = 0.02).

During asthma exacerbations, an additional nasopharyngeal swab was taken when participants (*N* = 63) were visiting the research facility for evaluation. A respiratory virus was detected in 54% of these swabs, with no differences between treatment groups (Figure S2; virus specification can be found in Table S3).

**FIGURE 4:**
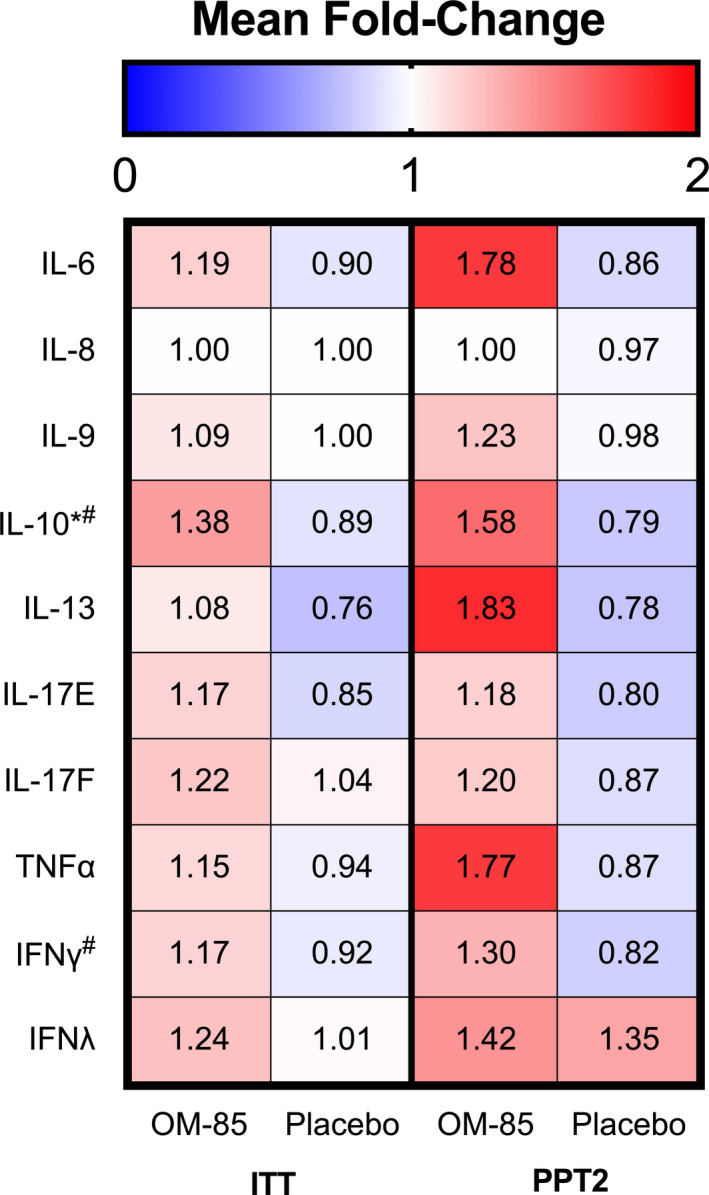
Plasma cytokine fold‐change ratios between baseline and 12 months for both the OM‐85 and placebo group (shown as mean fold‐changes). Significant differences (*p* < 0.005) after Mann‐Whitney *U* test shown as *for intention to treat (ITT) and #for patients with type 2 inflammation being adherent to the study protocol (PPT2)

No significant differences between treatment groups were observed for absolute numbers of blood eosinophils and neutrophils, serum IgG, IgA, IgM and IgE at baseline and during the 18‐month follow‐up (Table 3). IFNλ was higher at baseline within the OM‐85 group compared with placebo (20.78 [5.59;1156.00] vs. 5.59 [5.59;5.59] pg/ml, *p* = 0.01). No significant differences between the groups were observed within the other plasma cytokines at baseline. Linear mixed model analyses revealed that plasma cytokine levels of IL‐6 (*p* = 0.03), IL‐10 (*p* = 0.01), IL‐13 (*p* = 0.02) and IL‐17E (IL‐25) (*p* = 0.02) showed significantly higher levels in the OM‐85 group compared with placebo over time, which was not present in the placebo group (0 vs. 6 and 12 months, Table 3). This was reflected in a higher fold‐change ratio for plasma IL‐10 (*p* = 0.001) in the OM‐85 group as compared to placebo group (Figure 4).

### 3.4 Post hoc analysis; per protocol and type 2 inflammation

Thus far, our study could not show efficacy of OM‐85 on exacerbation frequency in the cohort of severe asthma patients. However, substantial clinical and immunological heterogeneity exists among asthma patients. The ITT analysis included patients that were not compliant with the study regimen or did not reach the primary end‐point in terms of time. PP analysis (OM‐85 *N* = 27; placebo *N* = 31) showed a slight but not statistically significant decrease in exacerbations (OM‐85 41 (1.52 ± 1.37) vs. placebo 61 (1.97 ± 1.89), IRR 0.77 (0.47–1.17), *p* = 0.23). We performed a parallel analysis on the PPT2 group (OM‐85 *N* = 22; placebo *N* = 20; both groups were comparable in terms of age, gender, BMI and asthma severity). Within 18 months, a reduction was seen in exacerbation frequency (30 OM‐85 vs. 48 placebo group) that did not reach statistical significance (IRR 0.71, 95%CI [0.39–1.26], *p* = 0.25) (Figure 3b; IRR for separate 6‐month period is shown in Table S2). Time between first and second exacerbation was also non‐significantly longer for OM‐85 compared with the placebo group (Figure S1c,d). Between the OM‐85 and placebo groups, no significant differences were seen in the fraction of virus‐associated exacerbations (58% vs. 55%), or in the prescription of oral corticosteroids and antibiotics (data not shown). The FEV1% values increased by 5.38% within 18 months in the OM‐85 group compared with the placebo group (95%CI 0.67–10.09, *p* = 0.03, Figure 3d).

Similar to the ITT group, plasma cytokine levels in the PPT2 group showed significantly higher levels of IL‐6 (*p* = 0.02), IL‐10 (*p* = 0.001) and IL‐13 (*p* = 0.03) in time with the use of OM‐85 compared to placebo (linear mixed model analysis; Table 4). Fold‐change ratios between T0 and T12 were increased for IL‐10 (*p* = 0.0001) and IFNγ (*p* = 0.0005) in the OM‐85 group compared to placebo group (Figure 4).

**TABLE 4:** Secondary end‐points for patients with type 2 inflammation being adherent to the study protocol (*N* = 42)

	Linear Mixed Model			OM‐85			Placebo		
	ED^a^	95% CI	*p*	T0	T6	T12	T0	T6	T12
Clinical parameters									
ACQ	0.18	−0.34;0.71	0.49	1.96 ± 0.95	1.36 ± 0.89	1.24 ± 0.75	2.21 ± 1.19	1.02 ± 0.86	1.40 ± 0.96
AQLQ	0.08	−0.35;0.51	0.71	5.23 ± 0.88	5.67 ± 0.73	5.78 ± 0.66	4.97 ± 1.06	5.42 ± 0.81	5.39 ± 0.81
FEV1*	5.38	0.67;10.09	0.03	92.11 ± 14.92	101.22 ± 14.93	95.79 ± 15.48	87.91 ± 13.05	88.22 ± 15.21	87.16 ± 17.84
FeNO	0.01	−0.01;0.01	0.93	31.35 ± 27.47	31.16 ± 30.71	28.70 ± 23.63	42.77 ± 52.51	39.23 ± 42.69	41.41 ± 46.89
Blood and serum routine immunological measurements									
Eo	−0.09	−0.53;0.33	0.64	0.36 ± 0.36	0.35 ± 0.33	0.30 ± 0.33	0.33±0.36	0.29 ± 0.19	0.35 ± 0.31
Neutro	−0.06	−0.26;0.15	0.59	4.31 ± 1.84	3.94 ± 1.62	3.72 ± 1.33	4.66 ± 1.98	1.18 ± 1.13	4.13 ± 1.84
IgG (g/L)	0.08	−0.07;0.23	0.28	9.81 ± 1.96	9.93 ± 2.47	10.67 ± 2.46	9.77 ± 2.55	9.79 ± 2.66	9.71 ± 2.83
IgA (g/L)	0.31	−0.04;0.65	0.08	1.89 ± 1.11	1.06 ± 1.15	2.21 ± 1.27	1.89 ± 0.99	2.01 ± 1.45	1.86 ± 1.11
IgM (g/L)	0.27	−0.02;0.56	0.07	1.32 ± 0.67	1.72 ± 2.18	1.27 ± 0.50	1.48 ± 1.91	1.12 ± 0.74	1.09 ± 0.69
IgE (kU/L)	0.63	−0.48;1.74	0.26	437.30 ± 531.95	419.25 ± 491.17	472.85 ± 124.50	579.77 ± 981.01	560.86 ± 922.41	605.91 ± 1030.30
Plasma cytokine measurements by ELISA (pg/ml)									
IL‐6*	0.99	0.19;1.79	0.02	0.77 [0.77–14.38]	0.77 [0.77–12.78]	3.12 [0.77–23.41]	1.03 [0.77–3.92]	0.77 [0.77–2.78]	0.77 [0.89–2.24]
IL‐8	0.14	−0.24;0.53	0.46	1.40 [1.40–1.40]	1.40 [1.40–1.40]	1.40 [1.40–1.40]	1.40 [1.40–1.40]	1.40 [1.40–1.40]	1.40 [1.40–1.40]
IL‐9	0.40	−0.07;0.87	0.09	2.21 [2.21–4.57]	2.21 [2.21–10.89]	2.21 [2.21–6.97]	2.21 [2.21–2.21]	2.21 [2.21–2.21]	2.21 [2.21–2.21]
IL‐10*	0.55	0.23;0.87	0.001	5.13 [1.40–29.41]	4.57 [1.40–36.54]	8.13 [1.40–56.58]	1.40 [1.40–9.14]	1.40 [1.40–7.95]	1.40 [1.40–3.96]
IL‐13*	0.71	0.06;1.35	0.03	144.36 [6.85–1381.80]	291.18 [38.96–1364.10]	504.53 [86.77–1393.50]	6.85 [6.85–523.57]]	51.76 [6.85–530.56]	6.85 [6.85–299.59]
IL‐17E	0.42	−0.07;0.92	0.09	20.23 [12.07–35.89]	14.35 [8.63–48.98]	20.86 [9.68–38.23]	6.98 [1.91–36.84]	4.42 [1.91–21.49]	2.82 [1.91–19.81]
IL‐17F	0.44	−0.13;1.01	0.13	33.64 [8.04–46.36]	23.84 [11.98–67.77]	24.18 [8.77–65.94]	9.79 [2.50–43.57]	6.69 [2.50–36.52]	7.89 [2.50–14.81]
IFNγ	0.64	−0.18;1.46	0.13	5.13 [1.89–8.83]	8.13 [1.82–10.80]	7.48 [3.61–11.40]	2.93 [1.53–9.81]	3.62 [1.53–10.61]	2.08 [1.53–5.91]
IFNλ	0.35	−0.04;0.75	0.07	40.74 [5.59–298.79]	59.75 [5.59–502.55]	135.75 [5.59–720.69]	5.59 [5.59–5.59]	5.59 [5.59–201.36]	5.59 [5.59–54.17]
TNFα	0.14	−0.79;1.09	0.76	2.79 [2.79–32.83]	2.79 [2.79–34.55]	12.79 [2.79–68.09]	2.79 [2.79–23.54]	2.79 [2.79–24.48]	2.79 [2.79–10.68]
PBMC flow cytometry (% OF CD4^+^ T‐lymphocytes or CD8^+^ T‐lymphocytes)									
CD4^+^ IL‐4	−0.09	−0.22;0.04	0.17	4.59 ± 1.91	5.21 ± 2.57	4.41 ± 1.81	4.49 ± 2.03	4.93 ± 1.61	5.03 ± 1.61
CD4^+^ IL‐5*	0.20	0.01;0.37	0.02	2.16 ± 1.19	2.16 ± 1.38	2.15 ± 1.20	1.82 ± 1.33	1.86 ± 1.23	1.75 ± 1.04
CD4^+^ IL‐9	0.03	−0.17;0.22	0.79	2.12 ± 0.59	2.38 ± 1.48	2.11 ± 0.71	1.98 ± 0.64	1.89 ± 0.69	2.01 ± 0.65
CD4^+^ IL‐10	0.15	−0.11;0.41	0.26	17.43 ± 12.64	3.93 ± 2.64	3.71 ± 2.47	3.51 ± 3.27	4.01 ± 3.36	4.46 ± 5.41
CD4^+^ IL‐13	−0.12	−0.32;0.09	0.26	2.60 ± 1.20	2.63 ± 1.48	2.61 ± 1.31	2.57 ± 1.33	2.55 ± 1.21	3.11 ± 1.50
CD4^+^ IL‐17A	−0.06	−0.26;0.14	0.56	1.19 ± 0.42	1.57 ± 1.12	1.45 ± 0.59	1.37 ± 0.66	1.27 ± 0.55	1.33 ± 0.49
CD4^+^ IFNγ	−0.08	−0.29;0.13	0.45	16.05 ± 12.37	14.89 ± 8.83	15.56 ± 11.77	17.43 ± 12.64	17.63 ± 8.70	18.59 ± 10.67
CD8^+^ IL‐4	0.12	−0.09;0.32	0.26	2.79 ± 1.41	3.15 ± 1.77	2.77 ± 1.26	3.16 ± 1.29	3.44 ± 1.61	3.05 ± 1.16
CD8^+^ IL‐5	−0.11	−0.29;0.06	0.21	2.26 ± 1.24	2.10 ± 1.44	2.32 ± 1.30	1.82 ± 1.33	1.86 ± 1.23	1.75 ± 1.04
CD8^+^ IL‐9	−0.02	−0.24;0.20	0.85	1.71 ± 0.59	2.02 ± 1.78	1.77 ± 0.56	1.55 ± 0.69	1.47 ± 0.66	1.35 ± 0.42
CD8^+^ IL‐10	0.13	−0.04;0.29	0.14	9.05 ± 5.98	9.05 ± 5.96	9.63 ± 5.73	6.43 ± 3.97	6.90 ± 3.84	6.81 ± 3.19
CD8^+^ IL‐13	0.19	−0.01;0.38	0.06	1.77 ± 0.89	1.77 ± 0.89	1.91 ± 0.99	1.80 ± 1.17	1.82 ± 1.14	1.93 ± 0.93
CD8^+^ IL‐17A	0.01	−0.22;0.22	0.99	0.91 ± 0.30	1.13 ± 0.78	1.09 ± 0.45	0.85 ± 0.30	0.87 ± 0.41	0.89 ± 0.32
CD8^+^ IFNγ	0.03	−0.14;0.19	0.70	39.28 ± 19.25	37.42 ± 12.67	38.44 ± 16.75	39.28 ± 19.25	37.42 ± 12.67	38.44 ± 16.75

Note

Data are shown as ED with 95% confidence intervals and its corresponding *p*‐value, mean values ± standard deviation or median [25^th^‐75^th^]. An estimate >0 correlates with a positive effect of OM‐85 on this variable.

Abbreviations: ACQ, asthma control questionnaire; AQLQ, asthma quality of life questionnaire; Eo, eosinophils (expressed as x10^9^/L); FEV1, forced expiratory volume in 1 second expressed as percentages; IFN, interferon (expressed as pg/ml); Ig, immunoglobulin (IgG, IgA and IgM expressed as gram/L, IgE expressed as IU/mL; IL, interleukin (expressed as pg/ml); Neutro, neutrophils (expressed as x10^9^/L); p, p‐value; TNF, tumour necrosis factor (expressed as pg/ml).

^a^ Estimated differences (ED) were calculated with a linear mixed model analysis. ED is the primary outcome of this analysis and stands for the treatment effect, or the ED in the independent variable between the OM‐85 and placebo treatment groups, adjusted for age, atopy and time.

* Significant difference.

Finally, flow cytometric analysis of intracellular cytokine levels in circulating CD4^+^ and CD8^+^ T cells in the PPT2 cohort did not show differences between the treatment groups over time (Table 4). The proportions of naive and memory CD4^+^ T cell subsets and B cell subsets did not differ between OM‐85 and placebo‐treated patients (Table S4). While proportions of CD86^+^regulatory T cells of CD4^+^ T cells showed a trend towards a significant increase over time, regulatory T cells did not show differences over time. Regulatory T cell activation, defined as proportions of naïve Tregs and active Tregs, also did not differ between the treatment groups over time (Table S4). Gating strategy can be found in Figure S3.

## 4 DISCUSSION

To our knowledge, this is the first study to describe the effects of add‐on bacterial lysate OM‐85 therapy in adult patients with severe asthma and recurrent exacerbations. We were unable to show a significant beneficial effect of OM‐85 on exacerbation frequency. However, we did observe a small positive effect on lung function as measured by FEV1% after OM‐85 treatment and found evidence for immune‐modulatory effects. Post hoc analysis, in the subgroup of 42 patients with type 2 inflammation adherent to the study protocol, revealed a non‐statistically significant trend for a clinically relevant reduction in exacerbation frequencies, in favour of OM‐85. This was observed especially during the 6 months after the intervention period, in this small subgroup of participants.

Liu et al.^30^ described a reduction in airway wall thickness and luminal stenosis in mice, after OM‐85 administration. Other studies suggest OM‐85 might reduce airway hyperresponsiveness and airway inflammation.^14^, ^15^, ^30^ Our findings confirm most studies performed on animal asthma models and suggest that bacterial lysate therapy may result in reduced airway hyperresponsiveness in human asthmatics reflected as a small increase in FEV1%. Confirmation in studies with a larger sample size is needed to ascertain these preliminary findings in humans.^31^

Previous studies in children showed that bacterial lysates were possibly effective in reducing asthma exacerbations.^18^, ^20^ Unfortunately, these studies were not all designed or powered to monitor exacerbation frequency. Studies in adults with COPD reported a decrease in exacerbation frequency with the use of bacterial lysates.^7^, ^32^ In our study, age was inserted as a covariate in the binomial regression model and was not statistically significant. We did not detect differences in the primary outcome in the younger age group <40 years of age. Childhood asthma is known to be mostly driven by allergic airway inflammation.^33^ Asthma in adulthood is more heterogeneous, with several endotypes identified according to the presence of allergies, eosinophilia, neutrophilia and obesity. Although ~60% of patients included in our study showed allergy‐driven asthma, the complete study group was small and heterogeneous with regard to the other asthma endotypes. As our post hoc analysis in patients with type 2 inflammation revealed a trend towards exacerbation reduction, we hypothesize that a longer treatment duration and/or different treatment regimen in a larger and more homogenous group of patients with type 2 inflammation may reveal a beneficial effect for OM‐85.

Several studies and meta‐analyses describe a reduction of recurrent RTIs with the use of OM‐85 in adults.^15^ In the self‐collected nasopharyngeal swabs, we did less frequently detect a virus in patients using OM‐85 during a clinical episode of RTI (OM‐85 30.5% vs. placebo 48.0%, *p* = 0.02), which is in accordance with previous literature. However, self‐reported RTIs are prone to subjective self‐reporting and could include episodes of allergic rhinitis. Nevertheless, we did not observe a difference in the numbers of viral RTI‐associated asthma exacerbations between patients using OM‐85 and placebo. Possibly, when having acquired a viral infection, immunomodulation by bacterial lysates in severe asthma patients does not prevent further progression to an asthma exacerbation.

Despite the absence of effects on exacerbation frequency in the complete study group, we did find evidence for immune‐modulatory effects induced by bacterial lysate therapy. These effects were mainly related to peripheral blood cytokine levels and lasted at least 6 months until after OM‐85 treatment (12‐month sampling time‐point). These effects seemed more pronounced in the PPT2 group as compared to the ITT group, with the former showing higher levels of several cytokines and especially IL‐10, a cytokine with immune‐modulatory functions and interferons. Some of these cytokines have been described as immune‐modulatory and anti‐inflammatory and can be produced by various immune cells including T cells. Flow cytometry analysis did not reveal significant changes in cytokine‐producing CD4^+^/CD8^+^ T cells, indicating that the observed increase in plasma cytokine levels might not be mediated by T cells. Previous observations in humans have also revealed higher levels of plasma IL‐10, after OM‐85 administration compared with placebo or control group.^17^, ^34^, ^35^ Also, local IL‐10 was shown to increase in the presence of CD86^+^ regulatory CD4^+^ T cells. IL‐10 might therefore be used as a biomarker for bacterial lysate‐induced immune‐modulatory responses. Future research in adults with asthma should address this possibility. Other cytokines increased in our study have a generally more pro‐inflammatory function, including cytokines associated with type 1 (IFNγ) and type 2 inflammation (IL‐13, IL‐17E). While the strongest effects were seen for IL‐10 and IFNγ, our study lacks evidence for suppression of type 2 inflammation as measured by levels of cytokines in the circulation.^36^

Our study did not show a significant effect of OM‐85 on function and numbers of naïve and activated Treg cells. In literature, animal models suggest an up‐regulation of CD86^+^ airway dendritic cells combined with an increase in Tregs and an up‐regulation of IL‐10 secreting cells in the trachea upon OM‐85 treatment. These up‐regulations result in a decrease in airway hyperresponsiveness and improved protection against airway inflammation in OM‐85 treated animals.^14^, ^30^ As we studied only systemic effects, it remains possible that different or stronger immunological effects can be found locally in the respiratory tract. Also, species‐specific differences might influence our outcomes.

Although carefully designed, our study has limitations. First, due to the unknown effect of OM‐85 in adults with asthma, we powered our cohort based on paediatric studies. Next, the number of asthma exacerbations during the study was lower than anticipated in the complete study group (mean number of 1.2 ± 1.4, as compared to 2.7 ± 1.2 before study start). Reasons for this could be a trial participation effect, as a result of the more frequent hospital visits or possibly increased therapy compliance and regression to the mean. This was observed in several other asthma studies and referred to as the Hawthorne effect. Nevertheless, our results suggest a potential clinically relevant beneficial effect for patients with type 2 inflammation, and therefore, this might be a good selection indicator as a starting point for future studies into the effect of bacterial lysate therapy. A different treatment regimen of OM‐85, which is currently being studied in the PrecISE network study (NCT04129931), might be more effective in adults.

In conclusion, our study did not demonstrate a benefit of bacterial lysate therapy on the incidence of asthma exacerbations in a heterogeneous group of adult patients with severe asthma and recurrent exacerbations. However, we did find an increase in FEV1% and non‐specific immune‐modulatory effects. Interestingly, post hoc subgroup analysis did show a trend towards a positive effect on exacerbation frequency in asthma with type 2 inflammation.

## CONFLICTS OF INTEREST

GT reports speaker fees and investigator‐initiated grant support from OM Pharma and Astra Zeneca, not paid in person but directly to a research foundation. GJB has received grant/research support for consultations and/or speaking at conferences from Novartis, GSK, AstraZeneca, ALK, Teva, Sanofi and Chiesi. GMB, EKP, CMZ, AB, GE, MN, JWB, GV, EB, BMBK, RS and RWH have no conflicts of interest.

## AUTHOR CONTRIBUTIONS

GT had the original idea for this study and designed the study protocol. GMB carried out the clinical part of the study under supervision of GT, GJB, GE and GV. GJB, GT, CMZ and AB assisted GMB with clinical follow‐up visits and sample collection. EKP, JWB and MvN assisted GMB with immunological analysis, EKP performed flow cytometric experiments under supervision of RWH and RS. GMB and GT performed statistical analysis under supervision of BBK and EB. GMB and GT wrote the manuscript, with special assistance on clinical topics from GJB and on immunological topics from RWH and RS. All authors reviewed the final manuscript and approved it before submission.

## ETHICAL APPROVAL

The study was approved by the national and local ethical review board and registered in the Dutch Trial Registry (NL5752). All participants provided written informed consent.

## Supporting information

Supplementary Material

Figure S1

## Data Availability

Data are available upon reasonable request via the corresponding author.
