# Supplementary material for: Bacterial lysate add‐on therapy to reduce exacerbations in severe asthma: A double‐blind placebo‐controlled trial
Source: Clin Exp Allergy. 2021 Aug 6;51(9):1172–84. doi: 10.1111/cea.13990 (PMC9292626; doi:10.1111/cea.13990)
Supplement: Supplementary file 1 — Supplementary Material [file CEA-51-1172-s002.docx]

**Supplementary documents**

**‘Bacterial lysate add-on therapy to reduce exacerbations in severe asthma:**

**a double-blind placebo-controlled trial**

**G.M. de Boer et al**

**1. Methods**

*Flow cytometry protocol*

Cells were extracellularly stained with antibodies for 30 minutes at 4°C and with LIVE/DEAD^TM^ Fixable aqua (Thermofisher) for 15 minutes at 4°C. For intracellular cytokine stainings, cells were stimulated with Phorbol 12-myristate 13-acetate (10ng/mL; Sigma) and ionomycin (500nM; Merck) in the presence of GolgiStop (BD) for 4 hours at 37°C. Cells were fixed with PFA (2%, 10 minutes, 4°C) and permeabilized with Saponin (0.5%, 30 minutes at room temperature). For analysis by flow cytometry, data were acquired on a symphony flow cytometer (BD) and analyzed with FlowJo (Tree Star Inc., USA) software (30).

**2. Figures**

**Supplementary Figure S1.** Kaplan-Meier curves of exacerbation-free survival.

Analysis of the intention to treat patient groups for **a**. time to 1st exacerbation (logrank p=0.610) and **b**. time between 1st and 2nd exacerbation (logrank= 0.560); Analysis of patients with type 2 inflammation adherent to the study protocol for **c**. time to 1st exacerbation (logrank p=0.599) and **d**. time between 1st and 2nd exacerbation (logrank p=0.220).

**Supplementary Figure S2.** Nasopharyngeal virus detection

Virus detection in nasopharyngeal swabs **a**. at baseline, after 3- and 6-months **b**. during asthma exacerbations. (Inner circle: placebo; outer circle OM-85).

**Supplementary Figure S3.** Gating strategy for CD4+ and CD8+T-cells and intracellular cytokines, regulatory T-cells and B-cells

**a.**

**b.**

**a.**


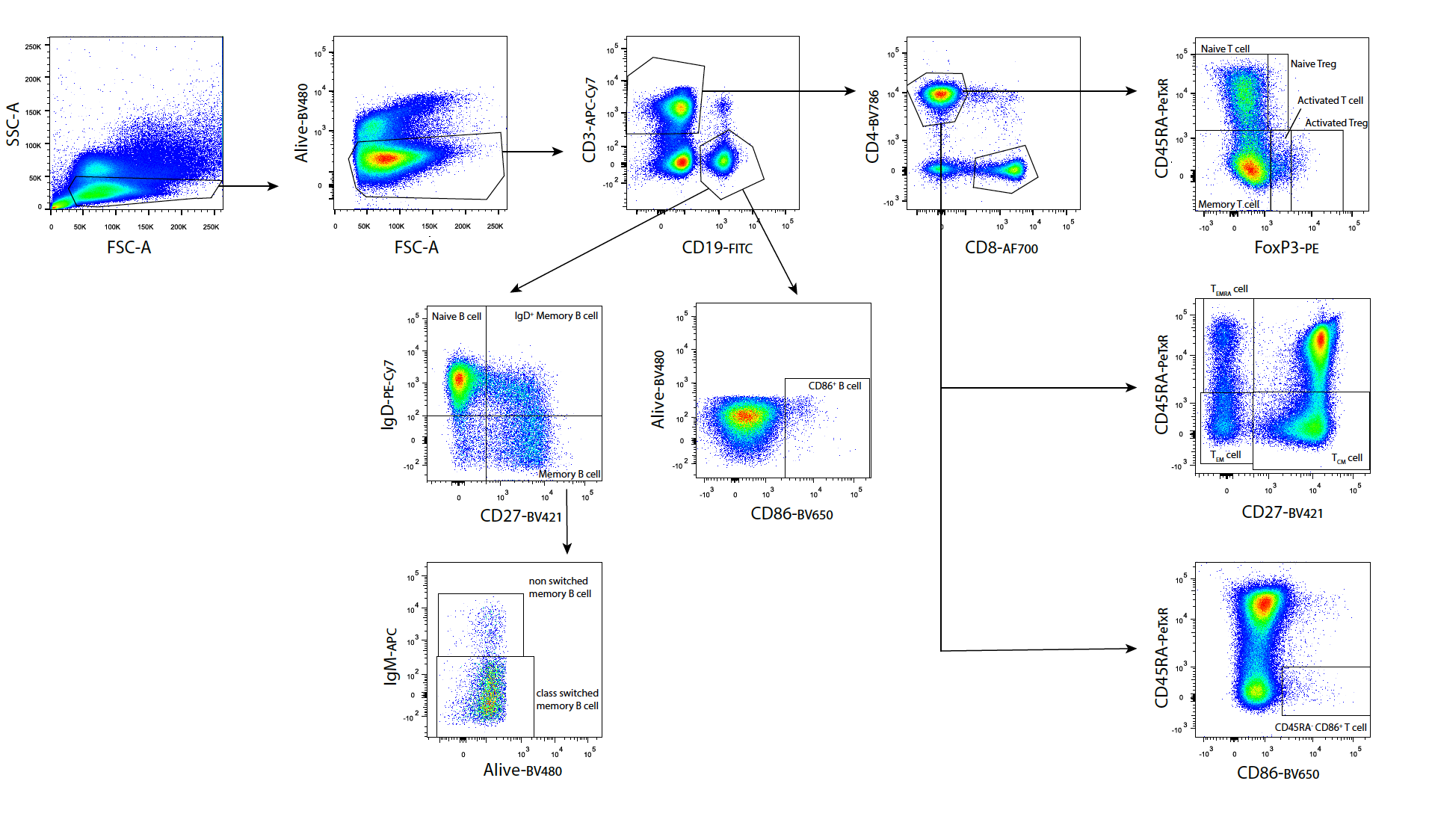


**b.**

**d.**

**c.**


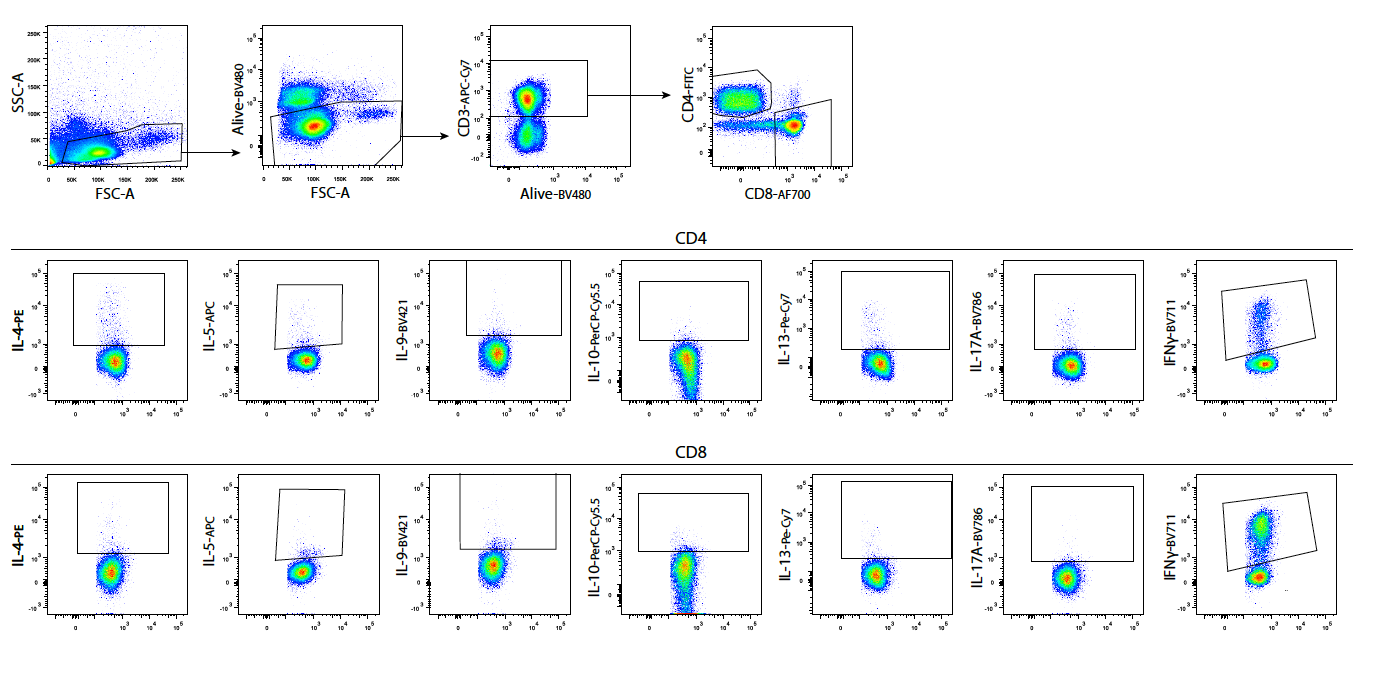


CD3-APC-Cy7

CD3-APC-Cy7

CD3-APC-Cy7

CD3-APC-Cy7

CD3-APC-Cy7

CD3-APC-Cy7

CD3-APC-Cy7

CD3-APC-Cy7

CD3-APC-Cy7

CD3-APC-Cy7

CD3-APC-Cy7

CD3-APC-Cy7

CD3-APC-Cy7

CD3-APC-Cy7

Flow cytometric gating strategy to identify **a:** CD4^+^ and CD8^+^ T-cell subsets, regulatory T-cells, B-cell subsets; **b:** intracellular cytokine levels in circulating CD4^+^ and CD8^+^ T-cells.

**3. Tables**

**Supplementary Table S1.** Antibodies used in flow cytometry analyses

| Marker *) | Clone | Channel | Company | Cat# |
| --- | --- | --- | --- | --- |
| IL-9 | MH9A3 | BV421 | BD | 564254 |
| IFNg | B27 | BV711 | BD | 564039 |
| IL-17a | N49-653 | BV786 | BD | 563745 |
| CD4 | SK3 | BV786 | BD | 563877 |
| CTLA4 | BNI3 | BV421 | BD | 562743 |
| CXCR3 | 1C6/CXCR3 | BV711 | BD | 563156 |
| CCR6 | 11A9 | APC | BD | 560619 |
| IL-13 | JES10-5A2 | PE-Cy7 | Biolegend | 501914 |
| IL-10 | JES3-9D7 | PCP | Biolegend | 501418 |
| IL-5 | TRFK5 | APC | Biolegend | 504306 |
| CD8 | SK1 | AF700 | Biolegend | 344724 |
| CCR4 | 205410 | FITC | R&D | FAB1567F-100 |
| IL-4 | 8D4-8 | PE | Thermofisher | 12-7049-42 |
| CD45RA | MEM-56 | PE TxR | Thermofisher | MHCD45RA17 |
| CD3 | UCHT1 | APC-Cy7 | Thermofisher | 47-0038-42 |
| FOXP3 | 236A/E7 | PE | Thermofisher | 12-4777-42 |
| CD4 | GK1.5 | FITC | Biolegend | 100406 |
| CD19 | HIB19 | FITC | BD | 555412 |
| IgD | IA6-2 | Pe-Cy7 | BD | 561314 |
| CD27 | M-T271 | BV421 | BD | 562513 |
| CD86 | 2331 (FUN-1) | BV650 | BD | 563412 |
| IgM | G20-127 | Bio | BD | 555781 |
| streptavidin | NVT | APC | BD | 554067 |

**Supplementary Table S2.** Incidence rate ratios (IRR) for the separate 6-month periods.

| Intention to treat | | | | Per protocol with T2 inflammation | | |
| --- | --- | --- | --- | --- | --- | --- |
| Study period | **OM-85 (N=38)** | **Placebo (N=37)** | **IRR (95% CI)** | **OM-85 (N=20)** | **Placebo (N=22)** | **IRR (95% CI)** |
| 0-6 months | 33 (0.9±1.0) | 26 (0.7±1.0) | 1.2 (0.7-2.1) | 17 (0.9±1.0) | 16 (0.7±0.9) | 1.0 (0.5-2.5) |
| 6-12 months | 13 (0.3±0.6) | 18 (0.5±0.7) | 0.7 (0.3-1.5) | 3 (0.15±0.49) | 15 (0.68±0.72) | 0.22 (0.1-0.8)* |
| 12-18 months | 25 (0.7±0.8) | 23 (0.6±0.8) | 1.0 (0.6-1.8) | 17 (0.77±0.81) | 10 (0.50±0.69) | 0.70 (0.3-1.6) |
| *Total* | 71 (1.9±1.7) | 67 (1.8±1.9) | 1.1 (0.7-1.7) | 30 (1.50±1.50) | 48 (2.18±1.94) | 0.71 (0.4-1.3) |

* p< 0.05

**Supplementary Table S3.** Viral and atypical pathogen presence in the nasopharynx during asthma exacerbations.

|  | OM-85 (N=38) | Placebo (N=37) |
| --- | --- | --- |
| Swab data available | **33** | **29** |
| No virus detected | **16 (48%)** | **12 (41%)** |
| Virus/atypical pathogen detected | **17 (52%)** | **17 (59%)** |
| Human rhinovirus | 7 | 1 |
| Coronavirus (OC43, NL63) | 2 | 4 |
| Influenzavirus A | 1 | 1 |
| Influenzavirus B | 0 | 1 |
| Respiratory syncytial virus | 1 | 4 |
| Human metapneumovirus | 3 | 1 |
| Parainfluenzavirus 1-3 | 2 | 2 |
| *Mycoplasma pneumoniae* | 1 | 1 |
| Adenovirus | 0 | 0 |
| Multiple viruses | 0 | 2 |

**Supplementary Table S4.** Trends in time between OM-85 and placebo for B- and T-cell subset proportions.

| B-subsets | | | |
| --- | --- | --- | --- |
|  | **Gating** | **ED (95% CI)** | **p-value** |
| % of lymphocytes | | | |
| B-cells | CD3^-^CD19^+^ | 0.03 (-0.13;0.19) | 0.72 |
| % of B-cells | | | |
| Memory B-cells | CD19^+^CD27^+^IgD^-^ | 0.31 (-0.04;0.65) | 0.08 |
| Naive B-cells | CD19^+^CD27^-^IgD^+^ | 0.03 (-0.03;0.08) | 0.36 |
| CD86^+^ B-cells | CD19^+^CD86^+^ | 0.02 (-0.23;0.27) | 0.87 |
| IgD^+^ memory B-cells | CD19^+^CD27^+^IgD^+^ | 0.02 (-0.14;0.17) | 0.84 |
| IgD^-^ CD27^-^ of B-cells | CD19^+^CD27^-^IgD^-^ | -0.03 (-0.12;0.06) | 0.53 |
|  | | | |
| T-subsets | | | |
|  | **Gating** | **Estimate (95% CI)** | **p-value** |
| % of lymphocytes | |  |  |
| T-cells | CD3^+^CD19^-^ | -2.46 (-9.65;4.72) | 0.49 |
| CD4^+^ T-cells | CD3^+^CD4^+^ | 1.02 (-6.60;8.65) | 0.79 |
| CD8^+^ T-cells | CD3^+^CD8^+^ | -1.60 (-8.43;5.23) | 0.64 |
| % of CD4^+^ T-cells | | | |
| Memory T-cells | CD3^+^CD4^+^CD45RA^-^FoxP3^-^ | 0.01 (0.11;0.13) | 0.92 |
| Naïve T-cells | CD3^+^CD4^+^CD45RA^+^FoxP3^-^ | -0.02 (-0.17;0.13) | 0.77 |
| Active T-cells | CD3^+^CD4^+^CD45RA^-^FoxP3^int^ | -0.14 (-0.38;0.09) | 0.24 |
| Treg | CD3^+^CD4^+^FoxP3^+^ | -0.13 (-0.37;0.11) | 0.29 |
| Naïve Treg | CD3^+^CD4^+^CD45RA^+^FoxP3^+^ | -0.14 (-0.39;0.10) | 0.25 |
| Active Treg | CD3^+^CD4^+^CD45RA^-^FoxP3^hi^ | -0.28 (-0.84;0.27) | 0.31 |

ED=Estimated difference (95 % CI) as analyzed by a linear mixed model.
