# Supplementary figures and images for: Bacterial lysate add‐on therapy to reduce exacerbations in severe asthma: A double‐blind placebo‐controlled trial
Source: Clin Exp Allergy. 2021 Aug 6;51(9):1172–84. doi: 10.1111/cea.13990 (PMC9292626; doi:10.1111/cea.13990)

**a.**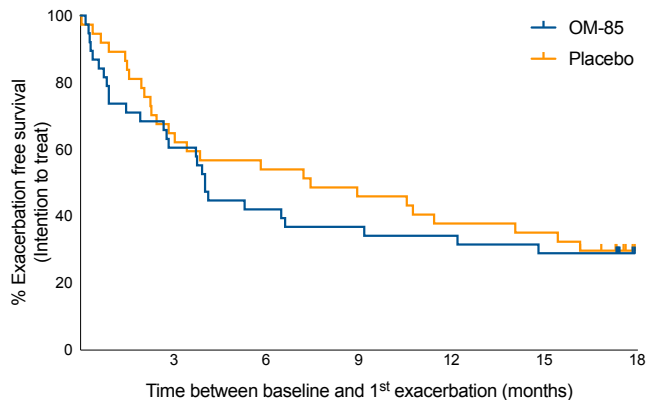**b.**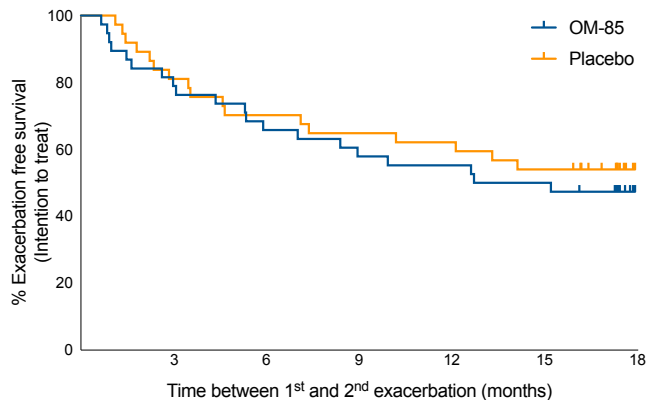**c.**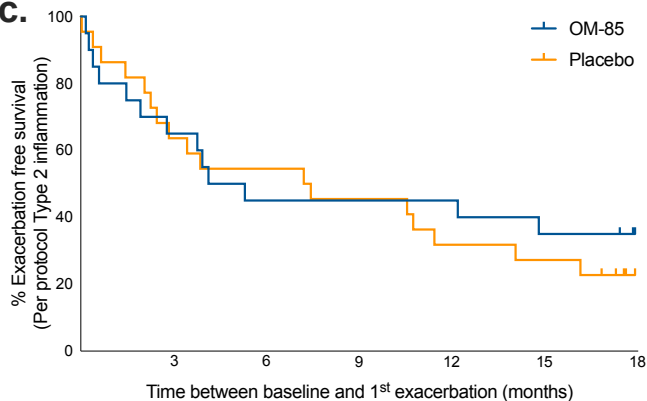**d.**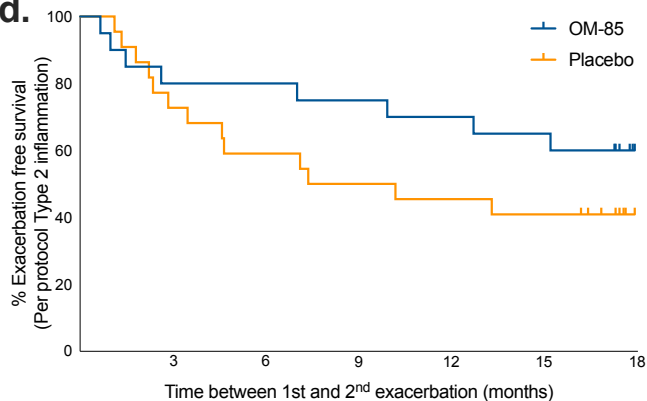

Supplement: Supplementary file 2 — Figure S1 [file CEA-51-1172-s001.pdf]
